# Who Is Affecting Who: The New Changes of Personal Influence in the Social Media Era

**DOI:** 10.3389/fpsyg.2022.899778

**Published:** 2022-05-25

**Authors:** Hongfa Yi, Yike Wang

**Affiliations:** ^1^School of Journalism and Communication, Shanghai University, Shanghai, China; ^2^School of Management, Shanghai University of International Business and Economics, Shanghai, China

**Keywords:** individual agenda, public agenda, opinion leaders, individual differences, topic modeling

## Abstract

With the development of social media, some individuals who have a great influence on the Internet have become opinion leaders, which means that the traditional agenda-setting theory cannot explain the mechanism of social consensus generation in the social media era. Therefore, the individual agenda is a new perspective to studying social consensus and personal influence in social media. This study defined the concept of the “individual agenda,” and conducted an empirical study on the relationship between the media agenda, the opinion leaders’ agenda, and the individual agenda, based on 71.77 million tweets sampled from the Twitter platform in 2015 with the approach of topic modeling. This study found that (1) most individual agendas are not consistent with the traditional public agenda, and the intrapersonal issue salience is highly related to the interpersonal issue salience; therefore, the concept of “individual agenda” has been validated empirically; (2) the media agenda has a significant positive correlation with 30.3% of the individual agendas, which means that professional media influences only a small number of individuals; and (3) the opinion leaders’ agenda has no significant correlation with the media agenda, while it has a significant positive correlation with 31.1% of the individual agendas, which means that opinion leaders have become strong competitors of traditional professional media in agenda-setting. This study also discussed the relationship between individual agenda-setting and public agenda-setting and the potential research directions in the future.

## Introduction

There are many problems that need attention in public social life, but which problems should be given priority? McCombs and Shaw (1972) put forward the concept of agenda-setting and considered that the audience’s understanding of the public issue salience usually comes from the news media (namely, the media agenda). Traditional agenda-setting studies summed up individuals’ understandings of the salience of public issues in the public agenda and compared the consistency between the public agenda and the media agenda, which made a significant contribution to explaining how media affect the attention of the audience in the old days.

However, with the development of social media, the suitability of the public agenda is gradually limited. On the one hand, social media has greatly enhanced the interaction between individuals (Hall, 2018). Thus, the issue salience may not only be reflected in the understanding of individuals but also in the interaction and discussion among them. On the other hand, the public agenda, as a summation of individual understandings, ignores individual differences, which are highlighted on social media. Too large individual differences may make the public agenda represent only a small part of the audience’s understanding of the issue salience, and a consensus on the issue salience will no longer exist. Thus, huge individual differences and strong interactions lead to a great diversity of public issues where some particularly active individuals gain a great influence and become opinion leaders (Veijalainen et al., 2015), who play an important role in agenda-setting in social media. Therefore, the important question that follows is: what role do opinion leaders on social media play in agenda-setting.

Therefore, agenda-setting studies on social media need to shift to the individual level (Guo, 2017), directly analyzing the relationship between the individual agenda, the media agenda, and the opinion leaders’ agenda. Such studies focus on the proportion of individuals who are significantly influenced by professional media or opinion leaders, rather than just measuring the relevance of the public agenda to the media agenda.

In this study, we defined the concept of the individual agenda and took 71.77 million tweets sampled in 2015 as a data resource, with the approach of text mining and topic modeling, to analyze the relationship between the media agenda, the opinion leaders’ agenda, and the individual agenda on Twitter.

## Theoretical Background and Hypothesis

### Public Agenda and Individual Differences

The public agenda-setting theory was proposed by McCombs and Shaw in the Chapel Hill study in 1968, which confirmed a significant correlation between the media agenda and the public agenda for the first time. However, the theory has not been without controversy since it was put forward. A core dispute is the measurement of the public agenda.

Dearing and Rogers (1996, p. 3) defined an issue as a social problem, often a conflictual one, that has been disclosed, and the agenda as a hierarchy of issue salience at a point in time. Thus, the media agenda is a hierarchy of issue salience in media coverage, and the public agenda is a hierarchy of issue salience among the public. As a concept at the aggregate level, the public issue salience cannot be directly measured; therefore, the traditional way is to first measure the salience of each individual’s issues and then add them together to obtain the public agenda (McCombs and Shaw, 1972). Because of this, Kosicki (1993) criticized the public agenda as a concept of measuring at the individual level but analyzing at the aggregate level; Becker (1991) questioned whether individual issue salience could be summed up into a public agenda.

The name of the public agenda misleads people into thinking that the public agenda is the overall agenda of the audience. In fact, the public agenda summed by individual issue salience is only an average of the individual agenda, which ignores individual differences and is not sufficiently representative of the entire public.

Assuming that individual issue salience conforms to a normal distribution, the public agenda, which is essentially an average of the individual agenda, lies at the mean point. Assuming that the public agenda is not significantly different from the issue salience of the individual within a unit. If the random variable *y* obeys a normal distribution with mathematical expectation μ and variance σ^2^, then the formula is denoted as *y* ∼ *N* (μ, σ^2^). When μ = 0 and σ^2^ = 1, *y* ∼ *N* (μ = 0, σ^2^ = 1) is the standard normal distribution. Under the standard normal distribution, the public agenda can represent most individuals (probability > 68.27%). When individual differences increase, such as when σ → 2, *y* ∼ *N*(μ = 0, σ^2^ = 4), the public agenda only represents 38.29% of individuals. If individual differences continue to grow, the public agenda can only represent a few individuals.

It is difficult for us now to re-examine the individual differences in the 1970s and 1980s. Maybe it is reasonable that agenda-setting studies at that time ignored individual differences and summed up the individual issue salience as the public agenda, because of the little difference in the traditional media era. However, with the rapid development of social media, individual differences have reached a level that researchers cannot ignore. Many scholars have noted individual differences in several studies, such as the long tail effect (Yang, 2013), individual heterogeneity (Hausman and Newey, 2016), audience segmentation (Purtle et al., 2018), media fragmentation (Riles et al., 2018), and individual legitimacy perceptions (Wang et al., 2020). In the social media era, a new understanding of the individual issue salience is that some social media users have a special interest in certain issues (McCombs et al., 2014). That is why the traditional public agenda-setting approach failed to reflect the consensus on social media.

### The Concept of Individual Agenda in the Social Media Era

McCombs and Shaw (1972) were aware of the defect that the study on public agenda-setting ignores individual differences, and they believed that follow-up studies should be transferred from the broad societal level to the social psychological level. Although later studies took individual differences into account, such as personal experience (Zucker, 1978) and the need for orientation (Camaj, 2014), they still remained in the analysis of aggregate level (Guo, 2017). Thus, real individual-level agenda-setting studies should be taken seriously, and the core concept of the individual agenda is emerging.

The term “individual agenda” has been mentioned in a few studies (e.g., Kosicki, 1993; Roessler, 1999; Arguelhes and Hartmann, 2017), but has not yet been clearly defined. Here, we define an individual agenda to be a hierarchy of individual issue salience (including intrapersonal issue salience and interpersonal issue salience), which means that each individual agenda has a ranking of issues and cannot be summed up to obtain an average.

In contrast to the public agenda that sums up individual issue salience, the individual agenda has no summation process, which means that the individual agenda appropriately reflects individual differences in issue salience. The individual issue salience in the public agenda is actually an intrapersonal salience that comes from intrapersonal cognition, that is, what a person believes to be the most important problem (MIP), regardless of what others say. This leads to a general questioning method (MIP) in agenda-setting studies to measure the intrapersonal issue salience (Yeager et al., 2011).

However, in the social media era, the issue salience of the individual agenda does not come exclusively from the intrapersonal issue salience. Interactive content between individual users on social media, which accounts for a considerable proportion of social media content, such as retweets and comments on the original content, is not intrapersonal cognition. Therefore, interactive content cannot be ignored and is taken into account in the study of the individual agenda.

McLeod et al. (1974) found that the individual issue salience contains two dimensions: intrapersonal issue salience and interpersonal issue salience, with sources coming from Lippmann and Park, respectively. According to Lippmann (1922), media, as a reflection of the outside world, influence people’s understanding of the world, which means that issue salience comes from personal cognition of the outside world. On the contrary, Park (1940) observes the media as part of a community that influences the discussion of issues reflected in the news, which means that issue salience comes from interpersonal discussion. Gadziala and Becker (1983) put forward a general questioning method to measure the interpersonal issue by asking, “Of all the problems facing the country, what did you discuss most with your friends last week?” McCombs (1977) had also made a clear distinction between the intrapersonal agenda and the interpersonal agenda; however, he insisted that the two cannot be added together for total issue salience because many issues that an individual considers important may not be present in his/her discussions with friends or family.

However, the difference between the intrapersonal agenda and the interpersonal agenda described by McCombs in the 1970s may be narrowing. The intrapersonal agenda and the interpersonal agenda have considerably overlapped because the interaction in social media is based on the original tweets; retweets and comments cannot exist on their own without the original tweets.

**FIGURE 1:**
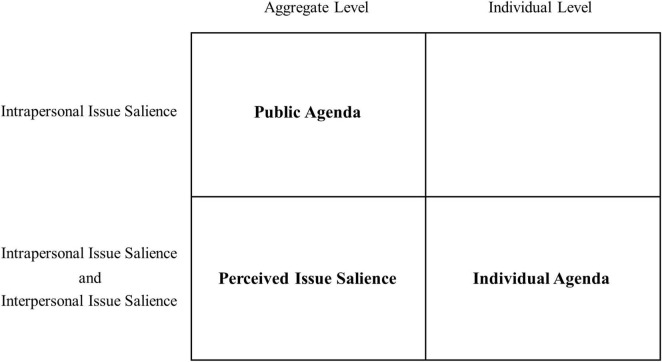
Concepts related to individual agenda.

As shown in Figure 1, the individual agenda is a concept at the individual level without a summation process and contains both intrapersonal and interpersonal issue salience. Perceived issue salience was proposed by McLeod et al. (1974) and also contains both intrapersonal and interpersonal dimensions, which are similar to the individual agenda; however, the measurement of perceived issue salience is still a summation process and is an aggregate level concept that cannot be equated to the individual agenda.

### Research Hypothesis

We have theoretically proposed and defined the concept of the individual agenda mentioned earlier, and in the following, we are going to test the rationality of this concept through an empirical study on Twitter. The two theoretical foundations of the concept of the individual agenda are individual differences and two-dimensional issue salience (intrapersonal and interpersonal), therefore the empirical study also addresses these aspects: (a) the expansion of individual differences in the social media era has led to the concept of public agenda no longer be suitable, and the public agenda is not representative of the majority of the individual agendas; and (b) the intrapersonal issue salience and interpersonal issue salience in the traditional media era are significantly different, thus the two cannot be added together; while the original tweets and interactive content, such as retweets and comments, in social media, are closely related, thus the two can be added together. Based on the above, we propose two hypotheses to verify the concept of individual agenda:

**H1:** Most individual agendas are not significantly correlated with the public agenda on Twitter.

**H2:** Intrapersonal issue salience is significantly correlated with interpersonal issue salience on Twitter.

Traditional agenda-setting research has focused on the influence of media on the audience; therefore, in this individual-level agenda-setting research, we also examine the relationship between the media agenda and individual agendas. Before that, we describe the distribution of the media agenda and the individual agenda on Twitter, such as the main issues presented by professional media and the main issues followed by individuals on Twitter. Existing agenda-setting studies generally view professional media as the influencer and individuals as being influenced (Vargo et al., 2014); therefore, this study will further examine the correlation between professional media and individuals on Twitter. If there is a significant positive correlation between the media agenda and the individual agenda, professional media are more likely to significantly influence individuals. Based on the above, we propose the research question:

Rq1: How many individual agendas have a significant positive correlation with the media agenda on Twitter?

In addition to professional media and ordinary individuals, there exists another important subject in social media: opinion leaders. The concept of opinion leader was first introduced by Lazarsfeld et al. (1960, p. xxiii) in their presidential election study, in which opinion leaders are more sensitive than others to the interests of their group, and more eager to express themselves on important issues. In social media, opinion leaders are those individuals who are particularly active and highly influential (Veijalainen et al., 2015).

In the examination of opinion leaders on Twitter, we assume that opinion leaders are likely to be dependents or competitors of professional media on Twitter. In addition to being able to influence the individual agenda, dependents are those whose agenda is highly similar to the media agenda, while competitors are those whose agenda differs significantly from the media agenda. In other words, the role of opinion leaders on Twitter depends on how the opinion leaders’ agenda relates to the media agenda and the individual agenda. Based on the above, we propose the research question:

Rq2: Is the opinion leaders’ agenda significantly correlated with the media agenda and the individual agenda on Twitter?

## Research Methods

### Data Source

The original data for this study were obtained through the Twitter API, whose openness has declined since 2016, therefore we conducted and completed data acquisition in March 2016. We first randomly selected 10 seed users and acquired a total of 4.06 million users through continuous iteration of users’ followers; then, we randomly selected 244,000 users at 6% and obtained all their 345 million tweets from March 2006 to March 2016.

We focus on 1 year of data to avoid too much dispersion of issues. Agenda-setting research primarily examines the relationship between agendas, rather than the frequency of specific issues. Therefore, the integrity of data is essential to this study. For this reason, we use the complete full year data in 2015, with a total of 71.77 million tweets.

The fields of each tweet contain two parts: text fields (e.g., tweet content, number of retweets, and number of likes) and user fields (e.g., username, user description, number of followers, and number of friends).

### Concept Measurement

The media agenda is measured as the content of media accounts that belong to professional media organizations with Twitter’s official verification, such as the Wall Street Journal, the New York Times, and The Washington Post. The measurement of the media agenda is primarily based on the number of media accounts reporting on a certain issue. We counted the frequency of each media account’s coverage of a certain issue, and then accumulated and ranked the data to obtain the media agenda. We measure the public agenda by first calculating the frequency of each individual’s original tweets on each certain issue, and then accumulating the frequency and ranking the data to obtain the public agenda. We measure the individual agenda by first calculating the frequency of each individual’s original tweets and the frequency of retweets and comments on each certain issue, and then adding the two frequencies to obtain the individual agenda.

The key to measuring the opinion leaders’ agenda is to identify the opinion leaders. In this study, user influence and user activeness were taken into consideration when identifying opinion leaders. The number of followers and retweets are the main indicators of user influence (Yerasani et al., 2019), and the number of tweets posted and the number of topics in which users participate are the main indicators of user activeness (Gu et al., 2016). In other words, opinion leaders are ordinary users who have an influence on Twitter, therefore we exclude media practitioners with Twitter’s official verification. Based on the abovementioned indicators, we took the top 5% of users and identified a total of 3,805 opinion leaders.

### Text Mining and Topic Modeling

The traditional content analysis and audience sampling survey are no longer suitable when it comes to individual agenda studies on social media. However, the development of big data text mining has made it possible to automate the analysis of large-scale texts. Topic modeling is a text mining technique applicable to agenda-setting research. The basic idea of topic modeling is that a text is a mixture of multiple topics, and a topic is a probability distribution of characteristic words. In other words, each text is a mixed distribution of topics, and each topic is a mixed distribution of a group of characteristic words (Yang and Zhang, 2018). Topic modeling has been used to identify topics in several studies (Grimmer, 2010; Bae et al., 2014), and according to Hong and Davison (2010), the accuracy rate of topic modeling on Twitter was up to 95.83%.

This study applies latent Dirichlet allocation (LDA) topic modeling (Martinez and Kak, 2001) to identify topics, the MongoDB data platform to store and query Twitter data, the GraphLab 2.1 (called by Python) to model 71.77 million tweets (the parameters of the LDA topic modeling: α = 0.1, β = 0.1, number of topics = 100; the solution method: Gibbs sampling; the number of iterations = 1,000). An issue is a public social problem, thus not all of the 100 topics obtained from topic modeling are issues. Therefore, we excluded (a) private problems, e.g., family relationships; (b) topics that do not make sense; and (c) content from non-media accounts and non-individual accounts, e.g., commercial advertisements and public service announcements. Finally, we removed unqualified topics from 100 topics and got 36 issues, including 1.53 million media tweets, 1.88 million opinion leaders’ tweets, and 20.92 million individual tweets.

The criteria for classifying and naming are based on two main considerations. For classification, we rely on the keyword distribution results of LDA topic modeling. For example, a topic identified by LDA topic modeling perhaps contains several keywords such as NFL, NBA, and football. In terms of naming, we refer to the existing literature on Twitter topic studies (Bantimaroudis and Zyglidopoulos, 2014; Wenner, 2014; Guo and Vargo, 2015; Rogstad, 2016). For example, according to the existing literature, we name the topic, including the keywords NFL, NBA, football, etc., as Sports.

## Results

### Descriptive Analysis

This study contains a total of 72,344 individual agendas, involving 20.92 million tweets. With most of the users (55,705), the top three issues they are most concerned about account for more than 30% of all their issues, which means that most users have a particular focus that reflects their individual differences.

As shown in Table 1, the public agenda involves 10.74 million tweets, and its top issue is Sports (7.0%). The media agenda involves 2,817 professional media accounts and 1.53 million tweets, and its top issue is also Sports (8.7%). The Pearson correlation coefficient between the media agenda and the public agenda is 0.62 (*p* < 0.001), which means that professional media are likely to set the agenda for individuals on Twitter from the perspective of public agenda-setting. The opinion leaders’ agenda involves 1.88 million tweets, and its top issue is video games (9.9%), but it only ranks 15th in the media agenda. The biggest difference between the opinion leaders’ agenda and the media agenda is the weather issue. Professional media pay less attention to the weather (0.9%), ranking last out of 36 issues, while opinion leaders pay more attention to the weather (5.3%), ranking fourth.

**TABLE 1:** Description of the agendas (frequency/percentage).

Issues	Public agenda	Media agenda	Opinion leaders’ agenda
Sports	746,571 (7.0)	132,958 (8.7)	107,626 (5.7)
Pop music	495,544 (4.6)	104,138 (6.8)	62,103 (3.3)
Education	247,303 (2.3)	89,206 (5.8)	54,137 (2.9)
Public security	277,566 (2.6)	74,413 (4.9)	39,919 (2.1)
Social media	706,866 (6.6)	72,619 (4.7)	118,370 (6.3)
Presidential election	531,873 (5.0)	66,733 (4.4)	97,852 (5.2)
Terrorism	211,526 (2.0)	62,941 (4.1)	40,541 (2.2)
Taxes	224,760 (2.1)	62,259 (4.1)	43,177 (2.3)
Transportation	567,630 (5.3)	51,163 (3.3)	96,430 (5.1)
Government activities	191,392 (1.8)	50,800 (3.3)	25,821 (1.4)
Racism	327,806 (3.1)	49,332 (3.2)	42,827 (2.3)
Political arguments	227,055 (2.1)	48,876 (3.2)	23,326 (1.2)
Government finance	213,855 (2.0)	48,119 (3.1)	22,654 (1.2)
Global trade	170,245 (1.6)	47,938 (3.1)	20,058 (1.1)
Video games	370,164 (3.5)	46,490 (3.0)	186,566 (9.9)
Business elites	214,518 (2.0)	44,254 (2.9)	23,174 (1.2)
Climate change	485,470 (4.5)	41,608 (2.7)	56,095 (3.0)
Space exploration	373,432 (3.5)	40,412 (2.6)	37,194 (2.0)
Nationalism	230,604 (2.2)	33,754 (2.2)	40,894 (2.2)
Natural disaster	202,565 (1.9)	31,630 (2.1)	28,386 (1.5)
Celebrities	193,125 (1.8)	31,314 (2.1)	19,910 (1.1)
Animal protection	311,937 (2.9)	25,988 (1.7)	70,560 (2.3)
Health	304,464 (2.8)	25,987 (1.7)	42,483 (3.7)
Entertainment	316,379 (3.0)	25,187 (1.7)	57,692 (3.1)
Energy	199,068 (1.9)	23,208 (1.5)	34,524 (1.8)
Medicine	202,813 (1.9)	22,321 (1.5)	40,867 (2.2)
Judiciary	149,770 (1.4)	21,558 (1.4)	28,503 (1.5)
Gender equality	198,190 (1.9)	20,741 (1.4)	33,333 (1.8)
Religions	266,375 (2.5)	20,257 (1.3)	52,096 (2.8)
Refugees	279,387 (2.6)	19,183 (1.3)	57,637 (3.1)
Urbanization	260,187 (2.4)	18,693 (1.2)	45,477 (2.4)
Mobile device	142,879 (1.3)	17,166 (1.1)	30,383 (1.6)
Crimes	174,233 (1.6)	16,648 (1.1)	30,493 (1.6)
Employment	205,854 (1.9)	16,310 (1.1)	47,754 (2.5)
Commercial Technology	157,844 (1.5)	13,752 (0.9)	28,980 (1.5)
Weather	360,347 (3.4)	13,014 (0.9)	99,220 (5.3)
Total	10,739,597 (100.0)	1,530,970 (100.0)	1,887,062 (100.0)

### The Empirical Test of the Individual Agenda Concept

**FIGURE 2:**
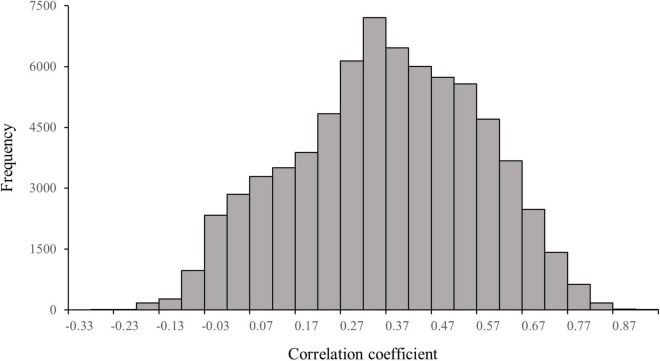
Distribution of correlation coefficients between the public agenda and the individual agenda on Twitter (*n* = 72,344).

As shown in Figure 2, the maximum value of the correlation coefficient between the public agenda and the 72,344 individual agendas is 0.87 (*p* < 0.05). The mean value of the 72,344 correlation coefficients is 0.27 and the median is 0.28, none of which reaches significance at α = 0.05 (the critical value for a significant positive correlation is 0.33). About 43,224 of the 72,344 individual agendas have a correlation coefficient of less than 0.33 with the public agenda, in other words, 59.7% of the individual agendas do not have a significant positive correlation with the public agenda.

In this study, intrapersonal issue salience involves 10.74 million original tweets and interpersonal issue salience involves 10.18 million retweets and comments, which are comparable in number, therefore the interpersonal issue salience cannot be ignored. The Pearson correlation coefficient for intrapersonal issue salience and interpersonal issue salience is 0.81 (*p* < 0.001), meaning that they are highly correlated.

### Relationship Between the Media Agenda and the Individual Agenda

**FIGURE 3:**
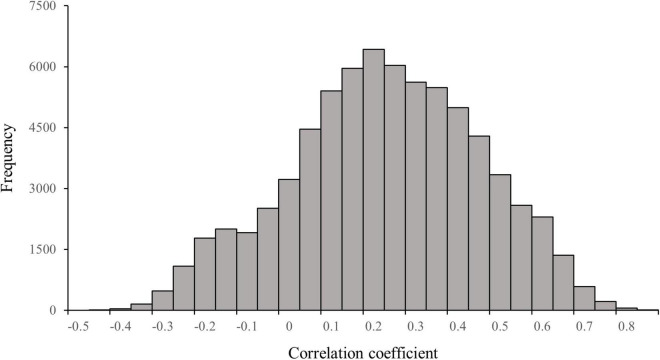
Distribution of correlation coefficients between the media agenda and the individual agenda on Twitter (*n* = 72,344).

As shown in Figure 3, 21,905 individuals have a correlation coefficient greater than 0.33 (critical value for a significant positive correlation) between their individual agendas and the media agenda, meaning that the media agenda was significantly and positively correlated with 30.3% of the individual agendas. The maximum value of the correlation coefficient is 0.84 (*p* < 0.001), which means that the professional media still has the ability to set the agenda for some of the ordinary individuals on Twitter. The minimum value of the correlation coefficient between the media agenda and the individual agenda is –0.50 (*p* < 0.01), which means that the professional media fails to set the agenda for a certain individual, and even this individual gives more salience to the issues that are less covered by the professional media. Of the 72,344 individuals, 13,223 (18.3%) show a negative correlation between their individual agendas and the media agenda on Twitter. The mean value of the correlation coefficient between the media agenda and the 72,344 individual agendas is 0.20, and the median is 0.21, neither of which reaches significance (*p* > 0.05).

### Relationship Between the Opinion Leaders’ Agenda, Media Agenda, and Individual Agenda

**FIGURE 4:**
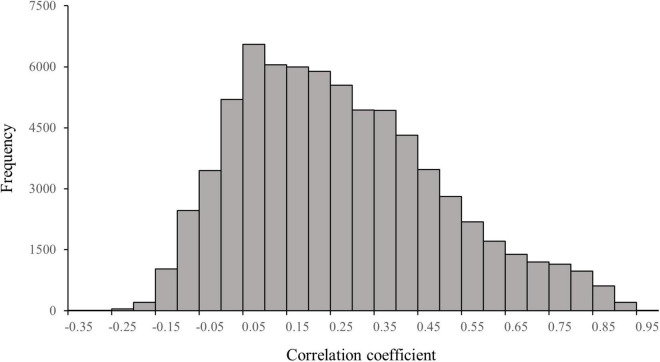
Distribution of correlation coefficients between the opinion leaders’ agenda and individual agenda on Twitter (*n* = 72,344).

As shown in Figure 4, the maximum value of the correlation coefficient between the opinion leaders’ agenda and individual agendas is 0.92 (*p* < 0.001), and the minimum value of the correlation coefficient is − 0.35 (*p* < 0.05) with a mean correlation coefficient of 0.22 and a median of 0.19. The correlation coefficient between 22,506 individual agendas and the opinion leaders’ agenda is greater than 0.33, meaning that the opinion leaders’ agenda may significantly influence 31.1% of the individual agendas.

The correlation coefficient between the opinion leaders’ agenda and the media agenda is 0.32 (*p* > 0.05), which does not reach significance. Therefore, there is a big difference between the opinion leaders’ agenda and the media agenda, and opinion leaders are unlikely to be dependent on professional media.

## Discussion

This study defined the concept of the individual agenda grounded in individual differences, and the new changes in the social media era, inheriting the legacy of Park, audience interactivity, emphasizing interpersonal issue salience.

### Research Findings

This study not only theoretically proposed the concept of individual agenda but also tested it by using the sample of data from the Twitter platform for the whole year 2015 (containing 71.77 million tweets). We verified H1: no significant positive correlation between the public agenda and the majority of individual agendas (59.7%); and H2: the Pearson correlation coefficient for intrapersonal issue salience (from original tweets) and interpersonal issue salience (from retweets and comments) reached 0.81 (*p* < 0.001), which means that individual differences and two-dimensional individual issue salience do exist in social media.

This study answered Rq1: the media agenda had a significant positive correlation with only 30.3% of the individual agendas on Twitter, which means that the media agenda can no longer influence the majority of individuals in the social media era. However, the media agenda remained significantly correlated with the public agenda (*r* = 0.62, *p* < 0.001).

This study also answered Rq2: the opinion leaders’ agenda was not significantly correlated with the media agenda, but was significantly and positively correlated with 31.1% of the individual agendas on Twitter. This answer indicated that opinion leaders are not dependent on professional media, distancing themselves from professional media on issues, developing their own content generated capabilities, becoming competitors, not dependents, of professional media, fighting for the agenda-setting power.

### Practical Implications

The introduction of the concept of individual agenda and the interpretation of the role of opinion leaders are new developments and changes in the agenda-setting theory in the social media era; however, this is not a complete rejection of the value of public agenda-setting research. Practically, individual agenda-setting may be a mechanism for achieving public agenda-setting, which continues to play an important role in public affairs decision-making.

Although the public may not have a consensus on what is an important issue, the government and related interest groups still need a ranking of issues, a twin to the public agenda, to facilitate decision-making, when addressing public issues.

This study found that the media agenda was able to influence 30.3% of individual agendas and had Pearson’s correlation coefficient of 0.62 (*p* < 0.001) with the public agenda. In fact, with 36 issues, the critical value of the Pearson correlation coefficient between the media agenda, the opinion leaders’ agenda, and the public agenda was about 0.33. Therefore, the media agenda and opinion leaders’ agenda only need to influence a very small amount of individual agendas (much less than 30.3%) to influence the public.

With opinion leaders becoming competitors for professional media and a decrease in the consensus on public issues, the government or related interest groups are able to influence the public whether through the media agenda, or opinion leaders’ agenda, which means that the public agenda is more likely to be influenced, and the difficulty of manipulating issues is substantially reduced.

### Limitations and Suggestions for Future Research

This study is limited to the Twitter platform, therefore, the generalizability of findings is limited. In the future, studies on individual agenda-setting should examine more social media platforms. This study used LDA topic modeling in text mining, and the accuracy of LDA algorithms determines the measurement accuracy of the main concept. Although existing LDA algorithms can achieve high accuracy (over 90%), errors may still lead to divergent findings. This study used data from a full year in 2015, and future studies could analyze data under other time units (months, weeks, and days) at a more granular level.

One direction for future research is the occasional conditions of individual agenda-setting (McCombs, 2014), and under what circumstances the media agenda is more likely to significantly influence the individual agenda. In comparison to the public agenda-setting research that examines the influence of media systems and issue attributes, individual agenda-setting research can better explain the effects of individual psychosocial factors on agenda-setting effects. Another direction for future research is to further clarify the role of opinion leaders in social media. This study found that opinion leaders are competitors for professional media, and further research can verify whether the role of competitors for opinion leaders is a normal state.

## Data Availability Statement

The raw data supporting the conclusions of this article will be made available by the authors, without undue reservation.

## Author Contributions

HY designed the study, analyzed the data, and drafted the manuscript. YW made a significant revision to the manuscript and assisted in data interpretation. Both authors contributed to the article and approved the submitted version.

## Conflict of Interest

The authors declare that the research was conducted in the absence of any commercial or financial relationships that could be construed as a potential conflict of interest.

## Publisher’s Note

All claims expressed in this article are solely those of the authors and do not necessarily represent those of their affiliated organizations, or those of the publisher, the editors and the reviewers. Any product that may be evaluated in this article, or claim that may be made by its manufacturer, is not guaranteed or endorsed by the publisher.
